# Rescripting Early Memories Linked to Negative Images in Social Phobia: A Pilot Study

**DOI:** 10.1016/j.beth.2007.04.003

**Published:** 2008-03

**Authors:** Jennifer Wild, Ann Hackmann, David M. Clark

**Affiliations:** a Institute of Psychiatry at King's College London; b University of Oxford

## Abstract

Negative self-images are a maintaining factor in social phobia. A retrospective study (Hackmann, A., Clark, D.M., McManus, F. (2000). Recurrent images and early memories in social phobia. *Behaviour Research and Therapy, 38*, 601–610) suggested that the images may be linked to early memories of unpleasant social experiences. This preliminary study assessed the therapeutic impact of rescripting such memories. Patients with social phobia (*N* = 11) attended 2 sessions, 1 week apart. The first was a control session in which their images and memories were discussed but not modified. The second was an experimental session in which cognitive restructuring followed by an imagery with rescripting procedure was used to contextualize and update the memories. No change was observed after the control session. The experimental session led to significant improvement in negative beliefs, image and memory distress and vividness, fear of negative evaluation, and anxiety in feared social situations. The results suggest that rescripting unpleasant memories linked to negative self-images may be a useful adjunct in the treatment of social phobia.

Individuals with social phobia often report experiencing negative, distorted images when in social situations. In their negative images they tend to see their worst fears being realized. Individuals with a fear of blushing, for example, may have images in which their face predominates and appears much larger and more flushed than it actually is. Clinically, such images appear to be problematic for a number of reasons. First, patients often believe that their negative images are an accurate reflection of how they appear to other people. They therefore think they come across much worse than they actually do, which tends to maintain their social anxiety. Second, the negative self-images seem to motivate patients to use self-protective strategies (safety behaviors) that are themselves problematic, such as covering one’s face to hide a blush or answering questions with one-word answers to avoid saying the wrong thing. Such behaviors prevent patients from disconfirming their fears (Salkovskis, 1991) and may also have the consequence of contaminating the social interaction by making patients appear unfriendly and aloof (Clark & Wells, 1995; Rapee & Heimberg, 1997).

One of the first empirical studies of imagery in social phobia was conducted by Hackmann, Surawy, and Clark (1998). Patients with social phobia and nonpatient controls were asked to recall a recent social situation in which they had felt anxious. They were then asked about any spontaneous imagery that may have occurred at the time. Almost all patients with social phobia reported experiencing negative images of themselves from the observer perspective. From this perspective, they saw their worst fears happening as if viewed from the outside. They also believed their images to be true at the time. Nonpatients were significantly less likely to report observer perspective images and, in addition, their images were less negative.

In a subsequent study, Hackmann et al. (2000) used a structured interview that aimed to further explore the nature of patients’ spontaneous images. All patients with social phobia reported that their negative, observer perspective images were recurrent in the sense that they tended to occur in different social situations. In addition, the images were linked in meaning and content to earlier unpleasant social events that occurred around the onset of the disorder. The images appeared to be extracted essences of memories of being criticized, humiliated, bullied, or experiencing other adverse social events. The authors hypothesized that early unpleasant memories lead patients to develop negative images of how they think they come across to others and these images are reactivated in subsequent social situations. Because the images are similar in content across social situations, it is suggested that they are not being updated in light of later, more benign experiences. This may be partly a consequence of excessive self-focus in social situations (Clark & Wells, 1995; Rapee & Heimberg, 1997).

Using a slightly different paradigm, four other studies (Coles, Turk, & Heimberg, 2002; Coles, Turk, Heimberg, & Fresco, 2001; Wells, Clark, & Ahmad, 1998; Wells & Papageorgiou, 1999) have investigated the perspective that patients with social phobia report taking when recalling social situations. Consistent with Hackmann et al.’s (1998) findings for spontaneous imagery, all four studies found that patients with social phobia were more likely than controls to take an observer perspective when recalling social events. This effect was largely confined to memories of social events (Wells et al., 1998), was more marked with high-anxiety events (Coles, Turk, et al., 2001), and became more marked as time since the event increased (Coles et al., 2002).

To date, three studies have experimentally manipulated negative self-imagery in individuals with social phobia or high social anxiety in order to determine whether it has a role in maintaining the disorder. All reported positive results. Hirsch, Clark, Mathews, and Williams (2003) asked patients with social phobia to have a conversation with a stranger while holding in mind either their usual negative image of themselves or a less negative (control) image. The negative image led participants to feel more anxious. They also thought their symptoms were more noticeable and that they had performed more poorly when they held the negative image in mind. Further, an assessor, who did not know which image participants held in mind, rated their anxiety as more evident and their behavior as less positive in the negative imagery condition. Thus, negative self-imagery increased anxiety and undermined effective social performance.

Vassilopoulos (2005) conducted a similar study with high and low socially anxious volunteers. Participants gave a speech in front of a camera. Half of each group held a negative observer perspective image during the speech, whereas the other half held a positive image of themselves. The high anxious group perceived more bodily sensations, rated specific aspects of their performance more poorly, and believed their self-image to be a more accurate reflection of how they came across when they held a negative image in mind.

Hirsch, Meynen, and Clark (2004) had high socially anxious individuals have two conversations with a conversational partner. During one conversation they held a negative image in mind and during the other, they held a less negative (control) image in mind. When holding the negative image in mind, the socially anxious volunteers felt more anxious. They also reported using more safety behaviors and believed that they performed more poorly. They also overestimated how poorly they came across compared to ratings the conversational partners made. Their partners rated them as performing more poorly in the negative imagery condition. This study replicated the earlier finding that negative imagery leads patients with social phobia to feel and look more anxious. In addition, it suggested that negative images motivate patients to use safety behaviors which can, in turn, contaminate the social interaction.

Recognizing the importance of negative self-images, several cognitive-behavioral treatment (CBT) programs for social phobia (for example, Clark & Wells, 1995; Clark et al., 2003; Heimberg & Becker, 2002; Rapee & Sanderson, 1998) include techniques for correcting distorted self-images. Until recently the techniques (video feedback, surveys of other people’s observations, behavioral experiments) have all been present-focused and have not attempted to directly modify the early memories that are linked to images. However, in a recent trial of cognitive therapy for social phobia (Clark et al., 2006), an imagery with rescripting technique was used to contextualize and update early memories of unpleasant social experiences in a subset of patients whose response to the standard, present-focused techniques was relatively modest. The authors speculated that use of the technique contributed to the good overall results observed in the trial but they were unable to provide data to support this speculation as the trial did not include a separate evaluation of the technique.

Imagery with rescripting techniques that focus on changing unpleasant memories have also been used as major components of CBT programs for borderline personality disorder (Giesen-Bloo et al., 2006) and for posttraumatic stress disorder arising from childhood sexual abuse (Smucker & Neiderdee, 1995). However, as with social phobia, the specific impact of the memory-focused techniques was not assessed.

As far as we are aware, the only published study that has attempted to isolate the specific impact of memory work is Ohanian’s (2001) single case report. After eight sessions of present-focused CBT, a 22-year-old woman with bulimia nervosa reported a 50% reduction in symptoms. Ohanian (2001) then looked at a critical early event that was linked to the patient’s most salient beliefs. To identify it, she asked the patient to describe an event in childhood in which she had negative feelings about herself. The rationale was that beliefs about her self-worth would have originated or been reinforced during a critical period. The patient identified an event that occurred when she was 10 years old. Imagery rescripting involved having her describe it in the present tense. At the point in the memory when she felt very hurt, she was encouraged to visualize her adult self entering the room to challenge the critical parent and then offer support and nurturance to her child self. This one session of memory work was followed by an almost complete cessation of binge-purge behaviors by the 3-month follow-up.

The present study was a preliminary attempt to identify whether imagery with rescripting focusing on early memories would generally be helpful in social phobia if given to an unselected group of patients in a tightly controlled fashion. It assessed the impact of rescripting early memories on negative images and the current symptoms of social phobia. We predicted that imagery with rescripting would reduce the strength of patients’ negative self-beliefs and their anxiety about feared social situations. We also anticipated a reduction in the frequency, vividness, and distress of patients’ recurrent images. By contrast, we predicted that simply exploring the memories would not be beneficial.

## Method

### Design

This study was conducted in a within-subjects, repeated-measures design. All participants attended a control session and then a rescripting session 1 week later. They completed measures before, after, and 1 week following each session. No other therapy was offered in this period.

### Participants

Eleven patients (seven female) who were receiving or about to receive cognitive therapy for social phobia were recruited. Seven were attending the Centre for Anxiety Disorders and Trauma in London and had started treatment (3 to 6 sessions). For these patients, the study was conducted during a gap in their therapy. Four had attended the University Psychiatry Department at the Warneford Hospital in Oxford for assessment and had not yet started treatment. Five patients had previously had emotion-focused counseling, one patient had attended 3 sessions of CBT for social phobia many years earlier, and five patients had never received previous treatment. All met criteria for social phobia on the Structured Clinical Interview for *DSM-IV* Axis I (SCID; First, Spitzer, Gibbons, & Williams, 1995) and the social phobia module of the Anxiety Disorders Interview Schedule (ADIS; Brown, DiNardo, & Barlow, 1994). Participants had a mean score of 24.00 (*SD* = 5.59) on the Fear of Negative Evaluation Scale (FNE; Watson & Friend, 1969) and 67.27 (*SD* = 31.28) on the Liebowitz Social Anxiety Scale—Self-Report version (LSAS-SR; Fresco et al., 2001). Their mean score on the Beck Depression Inventory (BDI; Beck, Rush, Shaw, & Emery, 1979) was 12.09 (*SD* = 12.46). Their mean age was 35.18 (*SD* = 9.36). The mean age of onset of the disorder was 16.27 years (*SD* = 11.86). The mean age at which the memory occurred was 17.09 years (*SD* = 13.41).

### Imagery interview

A semistructured interview (Hackmann et al., 2000) was administered immediately prior to the control session to elicit the description and meaning of participants’ recurrent imagery in social situations. This lasted approximately 30 minutes and asked a series of standardized questions in a fixed order. To introduce the interview, participants were told: “I’d like to talk to you about some of the things that go through your mind when you get anxious in social situations. Usually when people are very anxious a mixture of thoughts and images or fleeting pictures go through their minds. I’m especially interested in any pictures or images you have popping into your mind when you’re anxious. Do you have any spontaneous images when you are anxious in social situations?” All participants said that they did have images. They were asked to close their eyes and to recreate the image, then describe it. They were asked if this image recurred in social situations. All participants identified their image as recurring across social situations. To determine the meaning of the image, participants were asked: What is the worst thing about the image? What does it mean about you as a person? Participants were then asked to dwell on their image and to rate how vivid (real) it felt to them and how distressing it was (scales described below). They were also asked to rate how frequently the image had occurred in the previous week.

The interview then identified the memory linked to the image. To do this, participants were asked when they first remembered feeling the way they did in their image and to describe the event associated with that feeling. The interview probed for the meanings of their memory with similar questions that had been used to elicit the meaning of participants’ imagery. Participants were then asked to dwell on their memory and rate how distressing the memory was (scale described below). The interviewer summarized the meaning of the image and memory and asked participants to give one or two sentences that would “encapsulate” the meanings. One participant, for example, phrased the encapsulated belief meaning of her image and memory as “I’m an outsider and always will be. People will reject me or laugh at me because I’m not like them”. Her recurrent image was of looking awkward, jittery, twitchy, and speaking in garbled sentences. This was linked to a memory when she was 13 years old and a group of children at her school cornered her against a wall and made fun of her and the way she was twitching and unable to speak. She thought she would be attacked in front of all the other children and it would be humiliating.

### Measures

#### Encapsulated belief

This belief was elicited in the semistructured interview. As described above, it was a statement that captured both the meaning of the patient’s recurrent image and the linked early memory. Participants rated how much they believed this statement to be true on a scale ranging from 0 (*not at all*) to 100 (*extremely*).

#### Memory distress

Participants were asked to call their early memory to mind and to dwell on it. They were asked how distressing the memory was on a scale of 0 (*not at all*) to 100 (*extremely*).

#### Imagery ratings: Distress, vividness and frequency

Participants were asked to call their negative image to mind and to dwell on it. They were then asked how distressing the image was on a scale of 0 (*not at all*) to 100 (*extremely*). They were also asked how vivid it was on the same scale. Finally, they were asked how frequently the image had occurred in the previous week.

The above five scales are single-item rating scales and hence, there are no internal consistency data available. However, they have been used in a separate sample of patients with similar levels of social phobia (*N* = 14) and performed similarly. With the exception of image vividness, Pearson correlations of the association between the ratings before and after the control session in this study were moderate to strong, suggesting acceptable retest reliability: encapsulated belief (*r* = 0.60, *p* < 0.05), memory distress (*r* = 0.75, *p* = 0.01), image distress (*r* = 0.71, *p* < 0.01), vividness (*r* = 0.13, *p* < 0.71), and frequency (*r* = 0.92, *p* < 0.01).

#### Social anxiety

Participants completed the FNE and the LSAS-SR. The FNE is a self-report questionnaire concerning fears of negative social evaluation. It consists of 30 true-false statements. Higher scores indicate greater fear of negative evaluation. Examples of items include, “I am frequently afraid of other people noticing my shortcomings” and “I worry a lot about what my superiors will think of me.” The FNE is reliable. For example, Cronbach’s alpha was .92 when the questionnaire was administered to 150 undergraduates (Leary, 1983). The test-retest reliability was also good (*r* = 0.78) (Watson & Friend, 1969). The internal consistency of the FNE in our sample was excellent (α = 0.89). The FNE has good validity, discriminating between patients with social phobia and nonpatient controls as well as patients with other anxiety disorders (Turner, Beidel, & Larkin, 1986; Turner, McCanna, & Beidel, 1987; Stopa & Clark, 1993, 2000). The FNE correlates highly (*r* = 0.96) with the Brief FNE (Leary, 1983) that is currently more widely used.

The LSAS-SR is a 24-item scale measuring fear and avoidance in the past week of social and performance situations, such as giving a party, giving a report to a group, and trying to chat someone up. Participants were asked to rate how much fear or anxiety they had for each situation on a scale of 0 (*none*) to 3 (*severe*). They were also asked to rate how much they avoided that situation on a similar scale. The total score was derived by summing the two subscales. The LSAS-SR has good internal consistency. Among individuals with social phobia, Cronbach’s alpha for the total score was .95 (Fresco et al., 2001). In our sample, Cronbach’s alpha was .97. The LSAS-SR shows strong convergent and discriminant validity (Fresco et al., 2001).

In addition to completing the LSAS-SR, participants were asked to nominate their two most feared situations on this questionnaire and to imagine these scenarios with their eyes closed. They then rated how anxious each one made them feel on a scale of 0 (*not at all*) to 100 (*extremely*). They also rated how well they could picture each scenario in their mind’s eye on the same scale. The mean of the anxiety and picture ratings was calculated for both situations to give one score each: (1) Liebowitz anxiety score, which refers to how anxious the scenarios made them feel when they imagined them and (2) Liebowitz picture rating score, which refers to how well they could visualize the scenarios in their mind’s eye.

These rating scores, similar to the rating scales discussed above, are single item. There are no internal consistency data available for them. However, it was possible to gauge retest reliability. Pearson correlations of the association between the ratings before and after the control session were strong, suggesting good retest reliability: Liebowitz anxiety (*r* = 0.74, *p* < 0.01) and Liebowitz picture (*r* = 0.83, *p* < 0.01).

#### Presession measure

Participants completed the BDI prior to the control session. The BDI is a 21-item self-report questionnaire that measures the severity of depression in the previous week. Every item includes a group of four statements scored from 0 to 3, with higher numbers reflecting more severe depression. Participants were asked to read each group of statements and to circle the one statement that best described the way they had been feeling in the past week for the 21 items. The values of all 21 items were summed to give a total score. The BDI is reliable. Internal consistency in clinical (α = 0.86) and nonclinical samples (α = 0.81) is excellent (Beck, Steer, & Garbin, 1988). Coles, Gibb, and Heimberg (2001) reported that the retest reliability in patients with social phobia was strong (*r* = 0.84). Further, the BDI is a valid measure of depressive symptoms in psychiatric and normal samples (Beck et al., 1988; Bumberry, Oliver, & McClure, 1978; Kendall, Hollon, Beck, & Hammen, 1987). Participants completed the BDI once, prior to the control session. The internal consistency in our sample was excellent (α = 0.96).

### Interventions

The *control procedure* involved one and a half hours of exploring the early memory and the recurrent image. This was a nondirective session in which the therapist listened, reflected, and empathized but did not challenge or update the meaning of the early memory or recurrent image. Participants were encouraged to recall the early memory, to talk about how it made them feel, what it reminded them of, and whether they had other similar experiences in their lives.

The *memory rescripting* intervention built on Arntz and Weertman’s (1999) procedure in which patients revisit their memory in three stages. However, it differed in that it first involved cognitive restructuring. This was carried out for approximately 45 minutes prior to rescripting, which in itself lasted approximately 30 to 45 minutes. During the cognitive restructuring phase, the therapist and patient worked together to challenge the meaning of the early event and its implications for the present. For example, if a patient had been bullied and interpreted the event as meaning “I’m an outsider and always will be because I’m odd and different and weak; people will reject me or laugh at me if I am myself,” he was encouraged to come up with alternative ways of seeing the event. This would include thinking of all the reasons why children bully other children and what this says about the children who did the bullying, rather than him. He would also be encouraged to think of examples in which he was not rejected then or now. In essence, the therapist helped the patient to distinguish between what happened when he was a young child/teenager and what happens now as an adult in order to help him to see the event as a time-limited experience without implications for the present or future. The aim was to generate an adult perspective that the patient would then incorporate in the rescripting phase.

During memory rescripting, patients first imagined they were the age at which the event occurred and relived it as if it were happening again. Then they relived the memory at their current age, watching what happened to their younger self, and intervened if they wished, often conveying to the younger self the alternative perspective they had come up with in the cognitive restructuring phase. Finally, they relived it from the perspective of their younger self with their adult self in the room with them, intervening as before. This time the younger self was also asked what else he might need to happen in order to feel better, and the image then incorporated this material too. The younger self often requested extra nurturing and compassion at this point.

For each patient, the control and memory rescripting interventions were delivered by one of two clinical psychologists. Both had extensive training in cognitive therapy and had prior experience in rescripting memories linked to intrusive images.

### Procedure

The semistructured interview was administered to identify participants’ recurrent images, linked memories, and the encapsulated beliefs that covered both. The therapist then administered the control procedure. One week later, participants attended the rescripting session. A week after the rescripting session, a final set of measures was administered. Some measures were given at the beginning and end of the control and rescripting sessions. Some other measures were only given at the beginning of the control/rescripting sessions and 1 week later. Table 2 shows which measures were given at which time point.

## Results

### Within-Session Change: Encapsulated Belief, Memory Distress, and Anxiety About Social Situations

Due to the small sample size, we investigated within-session change by computing difference scores between measures taken at the beginning and end of the session and then performed *t*-tests to compare the magnitude of within-session change between the control and rescripting sessions. Difference scores were calculated for: (a) the encapsulated belief, (b) memory distress, (c) the Liebowitz anxiety score, and (d) the Liebowitz picture rating score. The ratings after the control and rescripting sessions were subtracted from the ratings taken at the beginning of these sessions. Table 1 shows the mean change in scores for the control and rescripting sessions. Compared to the control session, the rescripting session led to a significant reduction in how much participants believed their encapsulated belief, *t*(10) = 3.6, *p* < 0.01, and how distressing they found their early memory, *t*(8) = 4.5, *p* < 0.01. The rescripting session was also associated with a significantly greater reduction in participants’ anxiety ratings when visualizing their most feared social situations than the control session, *t*(10) = 3.5, *p* < 0.01. The two sessions did not differ in their effects on the vividness with which participants could visualize their most feared situations. *t*(10) = 1.9, *p* = 0.23, as indexed by the Liebowitz picture scale.

### One-Week Follow-up: Anxiety About Social Situations, Fear of Negative Evaluation, and Ratings for Imagery, Memory, and Encapsulated Belief

Repeated-measures analyses of variance with three levels (pre-control, follow-up control/pre-rescripting, and follow-up rescripting) were performed on measures of social anxiety and participants’ ratings of their intrusive images and encapsulated beliefs.

Table 2 shows the mean scores and standard deviations at the different time points, as well as the significance level of the paired contrasts. There was a significant effect of time for the encapsulated belief ratings, Liebowitz anxiety scores, and FNE scores. Paired contrasts showed that 1 week after the rescripting session, encapsulated belief, Liebowitz anxiety, and FNE scores had decreased significantly. In contrast, 1 week after the control session there was no change in encapsulated beliefs or social anxiety. The time effect for Liebowitz picture ratings was not significant, indicating that the two sessions did not differ in how well participants could visualize their most feared situations.

Turning to the image and memory ratings, there was a significant effect of time for the vividness of participants’ naturally occurring images and for the distress associated with those images. Paired contrasts showed that 1 week after the rescripting session, image distress and image vividness had significantly decreased. In contrast, 1 week after the control session, there was no change in either image distress or image vividness. There was also a significant effect of time for participants’ ratings of the distress associated with their memories. Paired contrasts showed that, 1 week after both the rescripting and the control sessions, memory distress had significantly decreased. The latter finding differs from the within-session analysis which found that the control procedure had no effect on memory distress. Finally, the time effect was not significant for image frequency, indicating that during the short period of the study, there was not a significant decline in the frequency of participants’ images.

## Discussion

This study is the first to investigate the therapeutic impact of rescripting social phobia–related traumatic memories. A control session in which patients simply explored their trauma memory led to no change in the meaning of the memory (encapsulated belief) and had no effect on either spontaneously occurring imagery or self-reported measures of social anxiety. In contrast, one session of memory rescripting produced significant within-session change in the meaning of the traumatic memory, the distress associated with the memory, and the amount of anxiety experienced when patients imagined participating in their two most feared social situations. In addition, 1 week after the memory rescripting session, patients reported that their spontaneously occurring images were less vivid and distressing. There was also a significant reduction in patients’ scores on the FNE, a standardized measure of cognition related to social phobia. Taken together, these results suggest that memory rescripting is an effective intervention in social phobia and may be worth considering as an adjunctive procedure in CBT programs. In the only treatment trial to date that has used the procedure (Clark et al., 2006), it was restricted to patients who had failed to show adequate response to more present-focused techniques. Our finding that memory restructuring was beneficial in a less restrictively selected group of patients suggests it may have broader applicability and might be usefully included as a routine procedure in cognitive therapy for social phobia. Future research will need to address the issue of the stage in therapy (early or late) at which rescripting might be most usefully introduced.

Memory rescripting has been a major component in some CBT programs for borderline personality disorder (Giesen-Bloo et al., 2006) and posttraumatic stress disorder arising from childhood sexual abuse (Smucker & Neiderdee, 1995). The finding that memory rescripting alone was effective in the present study suggests that it may have been an important contributor to the good outcomes obtained within those disorders. However, studies that isolate the effects of memory rescripting in the relevant disorders are needed to confirm this suggestion.

There are several possibilities as to how memory rescripting may exert its effect: it involves repeated evocation of the traumatic memory, cognitive restructuring, inserting new information derived from cognitive restructuring into the memory with an imagery exercise, and introducing a compassionate perspective, all of which may help the patient to reappraise the original event. In exposure treatment for posttraumatic stess disorder (PTSD), Foa and Rothbaum (1998) have shown that planned and controlled evocation of a traumatic memory can lead to its reevaluation. Although undoubtedly important, repeated evocation of the memory does not account for the results seen in this study, however. This is because both the control and rescripting sessions involved repeated evocation of the socially traumatic memory but improvements were only observed after the rescripting session.

The design of the study did not allow us to look at the separate contributions of cognitive restructuring, inserting new information into the memory, and a compassionate perspective. A future component analysis study will be required to definitively address this issue. However, the integrated procedure that was used in this study was the result of extensive pilot work with simpler interventions that only used some of the components and seemed less promising. We therefore suspect that each element played a useful role. The cognitive restructuring allows the patient to identify a convincing argument against the encapsulated belief. In our work with PTSD, we have found that intellectual shifts of this sort can be limited in their impact if the new information is not explicitly introduced into the trauma memory during a planned reliving (see Ehlers, Hackmann, & Michael, 2004, for an extended discussion), and the same may have applied here. In the compassionate imagery part of the intervention (Hackmann, 2005), the adult self revisited the traumatized self and was compassionate with affection and soothing words. This may enhance the patient’s feeling of being accepted, a central concept in social phobia.

One of the main effects of memory rescripting was that it reduced anxiety about feared social events. This may be a direct result of the reappraisal of the original event, leading to more adaptive, present relevant beliefs. However, patients also reported less vivid and distressing images after memory rescripting. Thus, it is possible that part of the reduction in social anxiety was mediated through changes in the nature of patients’ spontaneous images. This is consistent with previous research (e.g., Hirsch et al., 2003; Hirsch et al., 2004; Vassilopoulos, 2005) in which social phobics and high socially anxious individuals were less anxious having a conversation with a stranger when they held a neutral image in mind compared to when they held a negative image in mind. Considering Coles et al. (2001), it is also possible that the perspective of the memory changed with imagery rescripting. That is, patients may have experienced a more field and less observer perspective of their early memory, and this may have led them to feel less anxious about their usual feared situations. Perspective of the early memory was not measured in this study and would be a worthy focus of further research.

Contrary to hypothesis, there were no changes in the frequency of patients’ negative images following the rescripting session. As both the control and rescripting sessions were focused on the image and memory, patients were naturally more likely to be thinking about them in the week following their session. This may explain why there were no changes in frequency, despite the observed changes in vividness and distress. If this is correct, we would have expected to see a reduction in image frequency had we followed patients up for a longer period after the memory rescripting.

Perhaps the most intriguing finding in this study is the time in which patients experienced change. Change occurred within one session and it was maintained 1 week later. A possible reason for this quick change is that working in imagery is like having a concrete experience (Epstein, 1994). Lang (1977, 1979) suggests that the physiological, emotional, and behavioral responses activated during imagery are similar to what is activated in real scenarios. Drawing on research in neuroscience, we see that imagery of movement, for example of the hands, toes, or tongue, uses the same cortical circuitry (e.g., Schnitzler, Salenius, Salmelin, Jousmäki, & Hari, 1997) and results in the same motor cortical activation (e.g., Ehrsson, Geyer, & Naito, 2003) as actually moving these parts of the body. This suggests that at the level of brain activation, imagining movement is similar to actually doing it. Although imagery rescripting is much more than imagining movements, it is possible that the physiological, emotional, and behavioral responses it generates feel as real as actually having these experiences, and this may be therapeutic for clients. Further, imagery rescripting invokes many senses, and this may result in better activation of implicational meaning representations necessary to change them (Teasdale, 1993).

This pilot study has limitations. First, the sample size was small. Despite this, significant changes were seen in the rescripting session, which is encouraging. However, replication with a larger sample would be desirable. Second, it was not possible to counterbalance the order of the control and rescripting sessions. They were given in a fixed order with the control session offered first. This is because rescripting updated the original memory. Therefore, the control session would have had little material to explore had rescripting been offered first. Given the fixed order of sessions, we are unable to rule out the effects of the passage of time. Third, it is possible that there was a sequence effect in which giving the control session first made the rescripting session more effective. A future study could employ a between-subjects design in which groups are matched for symptom severity and one group receives rescripting, the other a control intervention. Fourth, we also used single-item rating scales in relation to imagery, memory, and belief ratings as well as anxiety and picture ratings of feared scenarios on the LSAS-SR. There are limited psychometric data on these measures and a future study would benefit from using well-established measures to assess imagery, memory, and anxiety in relation to feared scenarios. Nevertheless, preliminary analyses of these scales did suggest good retest reliability with the exception of image vividness. The assessment of retest reliability of the image vividness scale may reflect the lack of a relationship between how vividly patients experience their recurrent image before and 1 week after the control session, rather than poor retest reliability of the scale. Further studies are needed to assess the reliability and validity of these measures. Fifth, the small sample size meant that we were unable to use multivariate statistics to provide alpha correction for our use of multiple measures. Sixth, the study would have benefited from a longer follow-up period to track how long the changes last. This was not possible because the patients were all receiving cognitive therapy or about to start. Finally, this study relied exclusively on self-report measures of social anxiety (FNE and anxiety ratings for patients’ two most feared situations). A measure to assess behavior change, such as having a conversation with a stranger or giving a public talk, would be desirable. However, in their randomized controlled trial comparing cognitive therapy to exposure and applied relaxation, Clark et al. (2006) found that change on self-report measures of social phobia correlated with change on behavior tests. Therefore, it seems likely that the change on self-report measures of anxiety seen in this study following rescripting would also be associated with change on behavioral measures of social anxiety. Future studies could test this prediction.

## Figures and Tables

**Table 1:** Means (and standard deviations) of within-session change for the encapsulated belief, memory distress, Liebowitz anxiety and Liebowitz picture scores: Control versus rescripting sessions

Measure	Within-Session Change	
Control Session (*SD*)	Rescripting Session (*SD*)
Encapsulated Belief	11.36 (25.99)	52.95 (26.38)
Memory Distress	7.22 (12.28)	40.56 (22.14)
Liebowitz Anxiety	0.68 (11.84)	27.05 (22.02)
Liebowitz Picture	0.07 (29.10)	10.00 (16.51)

*Note*: Higher scores indicate greater improvement.

**Table 2:** Means and standard deviations at pre-session, post-session and 1-week follow-up on measures of social anxiety, imagery and memory

Measure	Pre-Control Session (1)		Post-Control Session (2)		Follow-up after Control Session/Pre-Rescripting Session (3)		Post-Rescripting Session (4)		Follow-up after Rescripting Session (5)		Analysis		Significance of Paired Comparisons	
Mean	*SD*	Mean	*SD*	Mean	*SD*	Mean	*SD*	Mean	*SD*	*F*	*df*	1 vs. 3	3 vs. 5
Liebowitz Anxiety	68.41	15.70	67.73	17.16	64.09	23.96	37.05	29.45	40.45	27.90	8.76⁎⁎	2, 20	0.538	0.009
Liebowitz Picture	67.66	21.00	69.20	19.59	71.81	16.44	61.81	20.00	57.05	25.47	2.73	2, 20	-	-
Encapsulated Belief	76.82	20.16	65.45	28.06	74.77	24.91	21.82	20.41	24.09	21.31	36.91⁎⁎⁎	2, 20	0.749	0.001
Memory Distress	68.18	27.14	60.56	28.22	53.18	26.48	8.89	15.37	22.27	19.92	17.28⁎⁎⁎	2, 20	0.025	0.001
FNE	24.00	5.59			23.91	5.56			17.91	10.26	6.25⁎	1.03, 10.34	0.863	0.041
Image Frequency	18.96	29.32			12.09	29.24			9.32	15.95	2.36	2, 20	-	-
Image Distress	50.00	26.55			47.73	27.78			19.55	25.54	8.40⁎⁎	2, 20	0.724	0.005
Image Vividness	60.91	23.75			51.82	29.01			26.82	33.45	5.36⁎	2, 20	0.410	0.015

*Note*. FNE = Fear of Negative Evaluation Scale. Repeated measures analyses of variance conducted with three levels (pre-control, follow-up control/pre-rescripting, and follow-up rescripting). Degrees of freedom in analysis of FNE adjusted for heterogeneiry of variance. ∗*p* < 0.05, ∗∗*p* < 0.01, ∗∗∗*p* < 0.001.
